# Inter-Organ Crosstalk in Neurodegenerative Disease

**DOI:** 10.3390/life15101499

**Published:** 2025-09-24

**Authors:** Elisabetta Carata, Moris Destino, Bernardetta Anna Tenuzzo, Elisa Panzarini

**Affiliations:** Department of Biological and Environmental Sciences and Technologies (DiSTeBA), University of Salento, 73100 Lecce, Italy; elisabetta.carata@unisalento.it (E.C.); moris.destino@unisalento.it (M.D.); bernardetta.tenuzzo@unisalento.it (B.A.T.)

**Keywords:** inter-organ crosstalk, gut–brain axis, muscle–brain axis, neuro-immune axis, neurodegenerative diseases, neuroinflammation

## Abstract

Inter-organ communication plays a vital role in the pathogenesis of neurodegenerative diseases (ND), including Alzheimer’s disease (AD), Parkinson’s disease (PD), and Amyotrophic Lateral Sclerosis (ALS). Emerging research highlights the involvement of the gut–brain axis, immune system, and peripheral metabolic systems in modulating neuroinflammation, protein misfolding, and neuronal dysfunction by releasing cytokines, adipokines, growth factors, and other soluble factors, which in turn affect neuronal health and systemic inflammation. This review explores the complex bidirectional interactions between the brain and peripheral organs, including the gut, adipose tissue, liver, muscle, bone and immune system. Notably, the gut microbiome’s role in neurodegenerative diseases through the gut–brain axis, the impact of adipose tissue in inflammation and metabolic regulation, and the muscle–brain axis with its neuroprotective myokines are also discussed. Additionally, we examine the neuro-immune axis, which mediates inflammatory responses and exacerbates neurodegeneration, and liver–brain axis that is implicated in regulating neuroinflammation and promoting disease progression. Dysregulation of inter-organ pathways contributes to the systemic manifestations of neurodegenerative diseases, offering insights into both potential biomarkers and therapeutic targets, and, in turn, promising strategies for preventing, diagnosing, and treating neurodegenerative diseases.

## 1. Introduction

Inter-organ communication is increasingly recognized as a critical factor in the pathogenesis and progression of neurodegenerative diseases (ND). While these disorders primarily manifest within the central nervous system (CNS), mounting evidence highlights the significant involvement of peripheral organs and their intricate bidirectional interactions with the brain that influence both the function of the central nervous system and the physiology of peripheral organs. This crosstalk relies on numerous axes, connecting the brain to muscles, liver, intestine, adipose tissue, and the immune system. These axes are not mutually exclusive and likely interact in a complex way to influence the onset and progression of several diseases, including neurodegenerative ones.

Potential axes involved in inter-organ communication in neurodegenerative diseases include the gut–brain axis, the brain–adipose tissue axis, the brain–liver axis, the brain-immune system axis, and the brain–muscle tissue axis [1]. In the gut–brain axis, the role played by microbiota and bone tissue is so important that the literature data reports a refinement of terminology to the microbiota–gut–brain axis and brain–gut–bone axis, respectively.

The first underscores the role of the gut microbiome in influencing brain health through the production of metabolites, neurotransmitters, and inflammatory mediators that can have systemic effects on neuronal function and contribute to neurodegenerative processes. The second refers to the possibility that hematopoietic stem cells migrate to the brain, gut, or bone, contributing to local inflammation and complex immune responses. The liver–brain axis is implicated in promoting neuroinflammation and altering the clearance of neurotoxic substances from the circulation due to hepatic dysfunction. Adipose tissue participates in inter-organ communication through the secretion of adipokines and inflammatory factors able to influence neurodegenerative pathways. The muscle–brain axis is increasingly recognized to exhibit neuroprotective effects and potentially can oppose neurodegenerative processes via myokines released during muscle contraction. Finally, the immune system serves as a critical mediator across these axes, as peripheral immune cells can infiltrate the CNS and ignite systemic inflammation, contributing to neuronal damage and disease progression.

**Figure 1:**
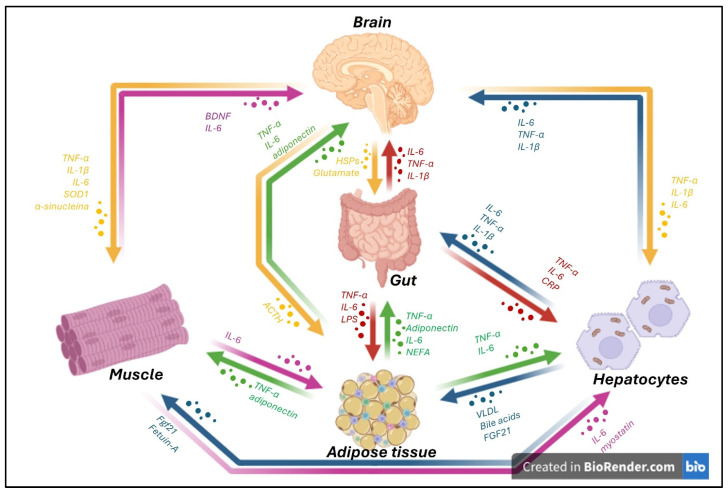
Schematic representation of inter-organ communication between key peripheral organs, such as the gut, adipose tissue, liver, muscle, and brain, mediated by the release of soluble factors and extracellular vesicles. In the presence of neurodegenerative diseases, the central nervous system (CNS) not only responds to peripheral inflammatory signals but also actively contributes to systemic inflammation through the secretion of pro-inflammatory mediators such as tumor necrosis factor-alpha (TNF-α), interleukin-6 (IL-6), and interleukin-1 beta (IL-1β). From the brain, cytokines including TNF-α, IL-6, and IL-1β, along with molecules such as superoxide dismutase 1 (SOD1), α-synuclein, and neurotrophic factors like brain-derived neurotrophic factor (BDNF), can influence distant tissues. For instance, these mediators affect skeletal muscle, where similar cytokines (TNF-α, IL-6, IL-1β) and stress signals are also produced. The gut plays a central role in this network, being both a source and target of inflammatory mediators. It releases TNF-α, IL-6, and IL-1β in response to microbial products (e.g., LPS) and communicates with the brain through molecules such as glutamate and heat shock proteins (HSPs). Adipose tissue acts as an important endocrine organ, producing adipokines like adiponectin, as well as TNF-α, IL-6, and non-esterified fatty acids (NEFAs), which modulate inflammation and metabolic signaling in the brain, muscle, and liver. Hepatocytes release cytokines such as TNF-α and IL-6, and acute-phase reactants like C-reactive protein (CRP), in response to systemic inflammatory signals. Conversely, they also secrete molecules like bile acids, Fibroblast growth factor 21 (FGF21), and Very Low-Density Lipoproteins (VLDL), which can influence metabolic activity and inflammatory tone in other tissues. Inter-organ signaling is further fine-tuned by reciprocal feedback loops. For example, muscle-derived myokines (e.g., myostatin) and the liver-derived hepatokine FGF21 influence adipose tissue and brain activity. Adrenocorticotropic Hormone (ACTH) from the brain also modulates peripheral inflammatory responses. Altogether, this complex network of cytokine-driven inter-organ signaling, particularly involving TNF-α, IL-6, and IL-1β, plays a critical role in the progression and systemic manifestation of neurodegenerative conditions, highlighting the importance of targeting peripheral inflammation in CNS diseases (the image was created with BioRender.com online software: https://www.biorender.com).

Communication between the organs involved in the different axes occurs through various mechanisms, including the release of soluble molecules, like cytokines, growth factors, metabolites, and hormones, or the release of extracellular vesicles containing packaged molecules [2] (Figure 1).

This review examines the emerging roles of the gut–brain axis, the immune system, and the peripheral metabolic system in influencing the main hallmarks, including neuroinflammation, protein misfolding, and neuronal dysfunction, of Alzheimer’s disease (AD), Parkinson’s disease (PD), and Amyotrophic Lateral Sclerosis (ALS). Dysregulation in the inter-organ crosstalk pathways can worsen disease pathology, contributing to systemic manifestations, and offer potential biomarkers to define therapeutic targets. At the same time, a deeper knowledge of the complex interplay between the brain and peripheral organs is crucial for developing new strategies to prevent, diagnose, and treat neurodegenerative diseases, also for the development of personalized medicine protocols.

## 2. Data Collection Methodology

Research articles and reviews focusing on inter-organ crosstalk involvement in neurodegenerative diseases’ pathophysiology were searched and chosen by using the Web of Science and PubMed databases. The first, containing more than 22,000 peer-reviewed journals, is one of the most complete and used database platforms. In the second is reported literature, including journals and books, from MEDLINE, the National Library of Medicine’s (NLM) premier bibliographic database that contains more than 31 million references in life sciences with a prevalence on biomedicine. For the collection of papers, we set a period from January 2021 to July 2025, and TS = (crosstalk) AND TS = (neurodegenerative diseases) AND TS = (immune axes) AND TS = (bioactive molecules) AND TS = (brain axes) as search terms and Boolean operators. Moreover, we have chosen to include manuscripts focused on the theme of brain-organs axes in neurodegenerative diseases, articles and reviews as document types, and only papers written in English; and to exclude themes not related to neurodegenerative diseases and briefings, news, meeting abstracts, etc., as document types.

## 3. Gut–Brain Axis

The gut–brain axis is an interconnected system encompassing neural, immune, endocrine, and microbial components that sustain an intricate interaction between the gastrointestinal tract and the nervous system [3]. This bidirectional communication occurs between the central nervous system (CNS) and the enteric nervous system (ENS), mediated by the autonomic nervous system (ANS), specifically by sympathetic and parasympathetic nerves. Each of these components comprises efferent cholinergic and noradrenergic fibers, as well as afferent sensory fibers, responsible for signaling between the brain and the gut, and vice versa [4]. The main function of the gut–brain axis is to monitor physiological homeostasis and to link the emotional and cognitive areas of the brain with peripheral intestinal functions and mechanisms, including immune activation, intestinal barrier permeability, enteric reflex, and enteroendocrine signaling [5]. The gut–brain axis includes the encephalon, spinal cord, ANS, the hypothalamus–pituitary–adrenal (HPA) axis and the gut microbiota [6]. The ANS is the portion of the peripheral nervous system largely responsible for the physiological and reflexive control of the body [7]. The gastrointestinal tract receives a dual extrinsic innervation: from the ANS, through cholinergic innervation from its parasympathetic division, comprising the vagal and pelvic nerves; and from noradrenergic innervation through sympathetic division, which consists of the splanchnic nerves. These two components provide the gastrointestinal tract with excitatory signals, stimulating intestinal activity, and inhibitory signals, reducing it, respectively [8]. In general, the ANS regulates various gastrointestinal functions, including intestinal motility and barrier integrity, luminal osmolarity, mucosal secretory responses, and immune responses [9]. While vagal and pelvic pathways primarily transmit non-painful stimuli to the brain, such as satiety, distension, and motility, the spinal splanchnic innervation is responsible for transmitting sensory information, including pain [8], upon interaction with metabolites derived from the intestinal microbiota [9]. This signaling is elaborated by the CNS, which responds by triggering an adaptive response with effects on peripheral target organs [9]. Langley was the first to hypothesize the existence of a third division of the ANS, termed the enteric nervous system (ENS) [10], located within the gastrointestinal tract, where it ensures homeostasis and performs numerous functions by interacting with the microbiota and host systems [9]. The ENS consists of a network of neurons intrinsic to the gastrointestinal tract and organized into two plexuses: the myenteric plexus, situated between the outer longitudinal and middle circular muscle layers, which controls intestinal motility, and the submucosal plexus, located between the middle circular layer and the mucosa, responsible for regulating secretion, blood flow, and nutrient absorption [11]. The ENS functions in an independent manner as it is able to regulate gastrointestinal activities and maintain homeostasis even in the absence of signals from the brain or spinal cord. It is due to the ENS structural and functional resemblance to the brain so that it is often referred as the “second brain” [12]. Consequently, dysfunctions within the CNS can be reflected in the ENS, and vice versa [9]. Furthermore, the synergy between the ENS, the intestinal epithelium, the immune system, the enteroendocrine system, and the gut microbiota is crucial as they ensure the absorption of nutrients, water, and electrolytes while simultaneously defending the organism against the entry of harmful substances into the intestinal lumen [12]. The vagus nerve establishes a crucial bidirectional flow of information between the brain and the gut through its interactions with the ENS [6]. The vagal innervation includes numerous organs such as lungs, liver, pancreas, spleen, ears, heart, and parts of the gastrointestinal tract such as the stomach and intestines [13]. Serving as a fundamental connection between the brain and the gastrointestinal tract, the vagus nerve’s key functions include the regulation of the activity of internal organs [6]. The vagus nerve contains both afferent and efferent fibers: approximately 80% of them are afferent, transmitting sensory information from the gut and other organs to the brain, encompassing taste, visceral, and somatic sensations. The remaining 20% of fibers are efferent, relaying signals from the brain to the gut, thereby regulating vital functions such as gastrointestinal, cardiac, and pulmonary activity [14]. Activation of the vagus nerve induces the release of acetylcholine, which by binding the α7 nicotinic acetylcholine receptors expressed on immune cells inhibits cytokine production. This mechanism has led to the concept of the “cholinergic anti-inflammatory pathway,” highlighting the vagus nerve’s important role as a neural brake on inflammation [13]. Recent studies have demonstrated that the nucleus of the solitary tract (NTS) is connected to regions involved in feeding behavior, specifically at the level of the lateral hypothalamus. It also interacts with neuropeptides such as orexin, which is important in gut–brain communication, and prolactin, implicated in modulating food reward and maintaining energy balance [13]. Orexin-immunoreactive neurons have been identified across all regions of the gut mucosa, with a predominant presence in the duodenum, as well as within the primary afferent neurons of the myenteric plexus in both animal models and humans [15,16]. The co-localization of orexin and its receptors on primary afferent neurons supports the existence of bidirectional orexinergic gut–brain communication, suggesting that peripheral orexin may detect nutrient availability and transmit signals concerning the organism’s nutritional status [15,16,17].

### 3.1. Microbiota–Gut–Brain Axis

Gut microbiota (GM) plays a pivotal role in the intricate system of the gut–brain axis, and researchers concur in the use of the term microbiota–gut–brain axis [18]. Comprising symbiotic bacteria that reside within the gut, GM is indispensable for maintaining gut homeostasis and supporting host health. These microorganisms are actively involved in crucial functions, including the metabolism of undigested nutrients, the production of beneficial metabolites, the defense against enteric pathogens, and the development of the immune system [19]. Given their active participation in host physiology, eukaryotic organisms are increasingly recognized as meta-organisms, functioning synergistically with their associated microbiota [20]. Emerging research highlights the significant influence of the GM on brain health and disease. Specifically, gut dysbiosis, characterized by an imbalance or alteration in the composition of the gut microbiota, is implicated in the pathogenesis of various neurodegenerative and psychiatric conditions. This contribution is believed to occur through multiple pathways, including the induction of oxidative stress, perturbations in energy metabolism, and mitochondrial dysfunction [21,22].

Interesting studies have reported significant differences in gut microbiota between healthy controls and AD patients, but the mechanisms of microbiome–brain interaction are so far unknown [23,24,25,26,27,28]. Gut microbiota can contribute to systemic inflammation and impaired physiological barriers due to the release of lipopolysaccharide (LPS), amyloid, and other toxins, which can travel from the gastrointestinal tract and oronasal cavity to the CNS. Bacterial amyloids have a mechanism of action like prion proteins, causing misfolding or aggregation of native amyloids. Furthermore, molecules released by the microbiota can activate microglia, promoting an inflammatory response in the CNS, which leads to increased neurotoxicity and reduced amyloid clearance. Given the role of Aβ as an antimicrobial peptide, infectious or sterile inflammatory factors can increase Aβ formation through toll-like receptors (TLRs). Modulating the composition of the gut microbiota may be used as a potential therapeutic target in AD [29].

In PD, the pathological roles of α-synuclein in gut–brain communication are the focus of numerous studies. Misfolded α-synuclein, a hallmark pathological feature of PD, may originate in the gut and subsequently propagate to the brain via the vagus nerve. This prion-like behavior of α-synuclein is thought to be exacerbated by intestinal dysbiosis and may be further compounded by the GM’s influence on intestinal inflammatory states and the integrity of the intestinal barrier. These processes collectively contribute to the formation and propagation of α-synuclein aggregates. Consequently, the gut–brain axis serves as a critical pathway in PD, with α-synuclein acting as a bridge between gut dysbiosis and neurodegeneration [30,31].

In ALS patients, the vagus nerve and enterochromaffin cells, which are involved in bidirectional communication between GM and enteric nerves, may play an essential role in the process of transmitting intestinal microbial signals to CNS. The vagus nerve may have long-term effects on the brain by activating chronic neuroinflammation, as a result of changes in GM. The enterochromaffin cells of the intestinal lumen can secrete substances such as 5-HT and histamine, which may activate the vagal afferent nerve endings as a consequence of stimulation from gut microbiota [32,33]. Similarly, in a mouse model, intestinal infection with *Campylobacter jejuni* can stimulate the vagus nerve, leading to the activation of the NTS or other visceral pathways related to the brain [34].

### 3.2. Brain–Gut–Bone Axis

**Figure 2:**
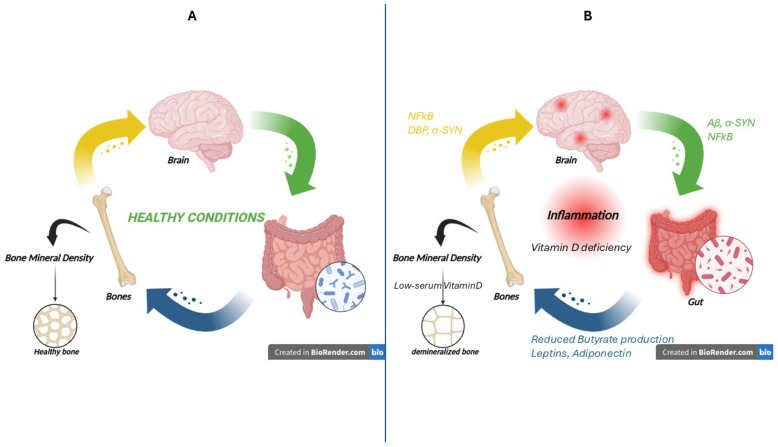
Bidirectional communication within the gut–bone–brain axis under physiological and inflammatory conditions. (**A**) In healthy conditions, balanced interactions between the brain, gut, and skeletal system preserve homeostasis. A healthy gut microbiota contributes to bone mineral density (BMD) and brain function through microbial metabolites and endocrine factors, sustaining bone integrity. Adequate serum vitamin D supports mineralization and prevents bone loss. (**B**) In inflammatory states, often associated with neurodegenerative disorders, this axis becomes disrupted. Vitamin D deficiency and dysbiosis reduce butyrate production and other adipokine secretion (e.g., leptin, adiponectin), which negatively affect bone turnover. The brain releases mediators such as nuclear factor kappa-light-chain-enhancer of activated B cells (NF-κB), vitamin D-binding protein (DBP), and α-synuclein (α-Syn), driving osteoclast hyperactivity and bone demineralization. Meanwhile, gut-derived factors, including Aβ, α-Syn, and NF-κB exacerbate neuroinflammation. This maladaptive feedback loop leads to decreased BMD, bone fragility, and sustained neuroinflammatory responses, underscoring the pathological role of the gut–brain–bone axis in neurodegeneration (the image was created with BioRender.com online software: https://www.biorender.com).

The brain–gut–bone axis represents an intricate network of bidirectional communication among the central nervous system, gastrointestinal tract, and skeletal system. This dynamic information is shared through diverse mechanisms, including neuronal pathways, immune system modulation, endocrine regulation, and metabolic interactions. The latest research suggests an intriguing role for this axis in the pathogenesis and progression of neurodegenerative diseases, proposing a complex relationship between dysregulation of the intestinal microecological environment and the skeletal system [35] (Figure 2).

Patients with AD are also considered to be at risk for osteoporosis as amyloid protein can be transported from the brain to peripheral organs and determine skeletal amyloid deposition, leading to the induction of osteoclast differentiation and activation upon interaction with the ligand of nuclear factor NF-kB receptor [36]. Commonly, vitamin D is essential for preventing osteoporosis because it increases the amount of calcium by acting on intestinal absorption from the food. Moreover, vitamin D is also involved in other physiological functions, including neuroprotection, the modulation of neuroinflammation, and the potentiation of glial- and brain-derived neurotrophic growth factors. In the context of AD, vitamin D affects the formation of beta-amyloid plaques and has a protective effect against neuroinflammation and its deficiency is associated with an increased risk of dementia and Alzheimer’s disease as neuroinflammation status, by leading to dysbiosis of the gut microbiota and to the consequent release of leptin and adiponectin, causes a reduction in the absorption of vitamin D and its precursors with consequent change in the density of bone [37].

Osteoporosis and osteopenia are common comorbidities also in PD patients, who frequently exhibit reduced serum levels of 25-hydroxyvitamin D (25(OH)D) compared to healthy controls. Elevated circulating levels of α-synuclein and the presence of Lewy bodies in both the midbrain and enteric neural terminals in PD patients led Figueroa and Rosen to propose that α-synuclein aggregation may alter the cellular dynamics of α-synuclein within osteocytes, thereby contributing to bone dysfunction in PD [38]. In this interplay, a crucial role is played by altered gut microbiota [39]. While the brain–gut–bone axis is recognized as a significant modulator in PD, its precise role and underlying mechanisms remain subjects of ongoing investigation.

During neurorehabilitation, ALS patients often exhibit alterations in bone health and a reduction in bone mineral density (BMD), as measured using bone mineral density and trabecular bone score assessments [40]. Some studies [41,42] suggest a direct link between low serum Vitamin D levels and reduced BMD, which may be associated with a less favorable prognosis in certain conditions. In the context of ALS, patients often experience nutritional deficiencies due to dysphagia (difficulty swallowing) and other feeding issues leading to a reduced intake and intestinal absorption of vitamin D. Additionally, limited sun exposure in these patients further exacerbates the risk of Vitamin D deficiency, thereby contributing to increase both bone fragility and risk of fractures [43]. Bone metabolism is also affected by the alteration of GM, particularly of butyrogenic bacteria. Studies have demonstrated that low levels of several butyrogenic bacteria, which are important for preserving intestinal integrity and modulating inflammation, are present in patients with ALS [44]. The relationship between butyrate and bone tissue is an active area of research and although the exact mechanisms are not fully elucidated, butyrate is thought to influence bone metabolism in several ways. Its anti-inflammatory action, by reducing systemic inflammation, may protect against bone mass loss, a key contributor to osteoporosis. Furthermore, some evidence suggests that butyrate may inhibit the activity of osteoclasts, the cells responsible for bone resorption, thus promoting a more favorable bone balance. ALS-susceptible superoxide dismutase 1 (SOD1) transgenic mice show that *Akkermansia muciniphila* may mitigate ALS symptoms, while *Ruminococcus* and *Desulfovibrio* species promote the disease [43]. Preclinical studies in ALS murine models of have demonstrated that butyrate administration can slow disease progression occurring through mechanisms of neuroprotection, modulation of inflammation, and the potentiation of neurotrophic factors, thereby helping to protect motor neurons from degeneration.

## 4. Liver–Brain Axis

The anatomical continuity between the intestine and the liver favors the interaction between the gut microbiota and the liver. In recent years, research has focused on the possible critical role of the liver in modulating neuroinflammation, suggesting that the liver-gut axis can participate in metabolic pathways such as immune response and detoxification [45,46]. The intricate interplay involving the brain and the liver, in which gut microbiota plays a pivotal role, so that researchers identify the liver-gut–brain axis as an important modulator in the pathogenesis and progression of several neurodegenerative conditions [47]. Dysregulation of this axis, often initiated by gut microbial dysbiosis or hepatic dysfunction, can lead to the production and systemic circulation of neurotoxic molecules. For instance, altered gut microbiota can produce a range of metabolites, such as short-chain fatty acids (SCFAs), bile acids, and lipopolysaccharides, that can significantly impact neuroinflammation. While SCFAs like butyrate can be neuroprotective by strengthening the blood–brain barrier, elevated levels of LPS from Gram-negative bacteria can promote an inflammatory cascade. The liver’s role is critical as it metabolizes these compounds; in liver dysfunction, these toxins may bypass detoxification and enter the systemic circulation. This allows molecules like LPS to cross the compromised blood–brain barrier, activating glial cells and leading to chronic neuroinflammation, oxidative stress, and the aggregation of pathological proteins [48].

**Figure 3:**
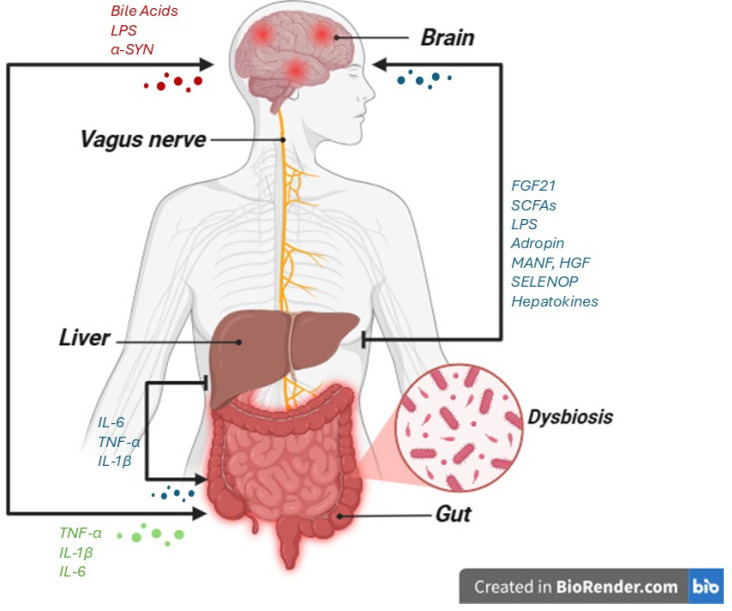
Role of molecules involved in inflammation along the liver-gut–brain axis in the presence of dysbiosis in neurodegenerative diseases. Intestinal dysbiosis impairs liver function, reducing detoxification capacity and promoting increased intestinal permeability (“leaky gut”), which facilitates the systemic translocation of endotoxins such as lipopolysaccharide (LPS). Bile acids are synthesized by the liver and regulated by the gut microbiota, acting as key mediators of inflammation and gut barrier integrity. Under pathological conditions, liver dysfunction and the accumulation of toxic molecules exacerbate neuroinflammation in the central nervous system, contributing to the progression of neurodegenerative diseases, including Alzheimer’s disease, Parkinson’s disease, and ALS (the image was created with BioRender.com online software: https://www.biorender.com).

It has recently been demonstrated that the liver produces endocrine moderators, defined hepatokines, which affect neurodegenerative disease. Additionally, some of these circulating factors transmit metabolic messages from the liver to the brain, thereby modulating body weight and food ingestion. Receptors for hepatokines have been identified in the CNS, while the precise neural targets and receptors for numerous hepatokines have yet to be fully elucidated [49]. Figure 3 reports the main signaling molecules involved in the liver-gut–brain axis.

The involvement of the liver functionality alteration in AD is due to its ability to moderate neuroinflammation as hepatocytes-released hepatokine-like proteins can cross the BBB and regulate nervous system function. For example, fibroblast growth factor 21 (FGF-21) influences the brain insulin sensitivity, whereas adropin (ApoJ) acts as a chaperone by binding unfolded proteins and, in the case of AD, it participates in both transport of beta amyloid and clearance. Further, the Mesencephalic Astrocyte-Derived Neurotrophic Factor (MANF) acts as a protective factor in cellular stress against tau hyperphosphorylation; finally, Selenoprotein P (SELENOP) is a protein content selenium, expressed in glial cells and involved in the sensibility to ferroptosis, whose low levels promote oxidative stress in the AD brain [50]. The Aβ metabolism is also modulated by liver bidirectional pathway 14,15-epoxyeicosatrienoic acid (14,15-EET) and epoxide hydrolase (sEH). In the liver, cytochrome P450 (CYP) enzymes produces 14,15-EET from arachidonic acid, which is then inactivated by soluble epoxide hydrolase (sEH) into a less active 14,15-dihydroxyeicosatrienoic acid (14,15-DHET), able to regulate brain Aβ metabolism [51].

As described in the previous paragraph, the alteration of the GM contributes to the progression of PD in association with other risk factors such as coffee consumption, smoking, brain trauma, and liver dysfunction [52]. An increase in αSyn expression and neuroinflammation is observed in mice after the transplantation of GM from PD patients [53]. Instead, neuroinflammation and inhibition of the TLR4/MyD88/NF-κB pathway are observed in PD mice transplanted with GM derived from healthy mice [54]. The alteration of GM modulates liver activity: liver releases hepatocyte growth factor (HGF) with neuroprotective effects, able to reduce the dopaminergic loss in PD animals, and promote anti-inflammatory response by TNF-α, and ERK1/2 [55].

In ALS, the progression of disease is associated with liver dysfunction and metabolic abnormalities, confirming the link between liver and neurodegeneration, as demonstrated by Zhu in ALS patients [56].

Compromised hepatic fatty acid metabolism and detoxification capacity may intensify systemic inflammation. The endotoxins can breach the compromised intestinal barrier, a phenomenon known as leaky gut, subsequently activating inflammatory cascades within both the liver and the CNS [57,58].

## 5. Neuro-Immune Axis

**Figure 4:**
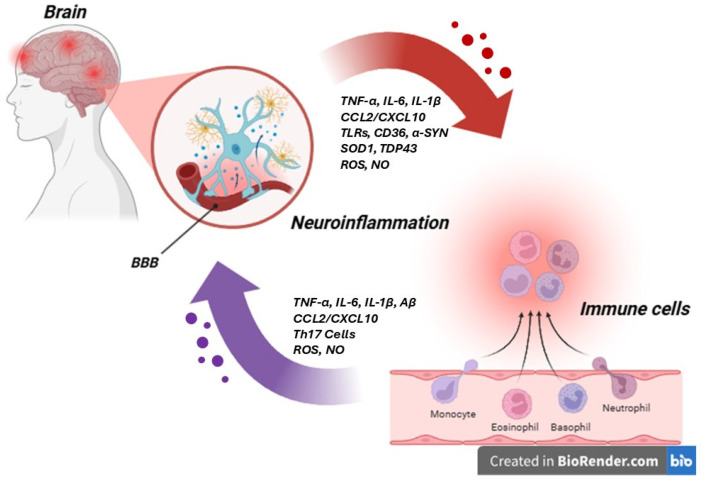
Schematic representation of the neuro–immune axis and its inflammatory mediators in neurodegenerative diseases. The bidirectional communication between the central nervous system and the peripheral immune system is mediated by a complex network of cytokines, chemokines, and danger-associated molecular patterns (DAMPs). In the CNS, activated microglia and astrocytes release pro-inflammatory cytokines, such as TNF-α, IL-1β, and IL-6, and chemokines like CCL2/MCP-1 and CXCL10, promoting neuroinflammation and recruiting peripheral immune cells. DAMPs such as amyloid-β and α-synuclein activate TLRs, NLRP3 inflammasomes, and receptors like CD36 and LAG3, further amplifying the inflammatory response. Infiltrating macrophages and T cells contribute to CNS inflammation through the release of ROS, NO, and cytokines. This molecular crosstalk is central to the pathogenesis of diseases like Alzheimer’s, Parkinson’s, and ALS, linking peripheral immune activation with central neurodegeneration (the image was created with BioRender.com online software: https://www.biorender.com).

The neuro-immune axis represents a critical and highly intricate bidirectional communication network between the nervous and immune systems, playing a pivotal role in maintaining physiological homeostasis, whose alteration leads to the initiation and progression of neurodegenerative diseases. This multifaceted axis involves not only intrinsic CNS cellular components, such as microglia, astrocytes, and oligodendrocytes, but also peripheral immune cells, including T cells, B cells, and macrophages, which can infiltrate the CNS under pathological conditions, inducing the leakage of the BBB. The communication within this axis is facilitated by a complex interplay of molecular mediators, including cytokines (e.g., TNF-α, IL-1β, IL-6), chemokines (e.g., CCL2, CXCL10), growth factors, hormones, neurotransmitters, and neuropeptides, alongside direct cellular interactions [59,60,61,62] (Figure 4). Dysregulation within this meticulously balanced system can disrupt CNS homeostasis, leading to amplified neuroinflammation, oxidative stress, proteinopathy (misfolding and aggregation of proteins), mitochondrial dysfunction, and ultimately neuronal dysfunction, damage, and death. Furthermore, alterations in the BBB integrity can facilitate the ingress of peripheral immune cells and inflammatory mediators into the CNS, exacerbating the pathological cascade [63].

In AD, the neuro-immune axis is centrally implicated in pathogenesis, characterized by the deposition of amyloid-β, which activates microglia and astrocytes, initiating an inflammatory response. Although microglia may initially phagocytose Aβ, their clearance function can be impaired in AD [64]. Activated microglia release various inflammatory mediators, such as TNF-α, IL-1β, and IL-6, which induce neuroinflammation and exacerbate neuronal damage, also by promoting aberrant tau protein hyperphosphorylation [65], also promoted by peripheral macrophages [66]. The activation of astrocytes can lead to glutamate metabolism disturbances and increased BBB permeability, facilitating the entry of inflammatory cells and mediators into the brain and contributing to neuroinflammation [67,68]. The activation of TLR by Aβ and tau upon inflammatory mediators released by infiltrated peripheral immune cells acts as DAMPs and promotes microglial and astrocytic activation and the release of inflammatory mediators. Moreover, oxidative stress, mitochondrial dysfunction, autophagy, and ubiquitin-proteasome system (UPS) anomalies contribute to the accumulation of abnormal proteins and neuronal inflammation, further intensifying AD pathology [63,69,70,71].

In PD, the neuro-immune axis is deeply implicated in pathogenesis, particularly through the aberrant aggregation of α-synuclein and the subsequent neuroinflammation [72]. In fact, pathogenic α-synuclein fibrils act as DAMPs, activating NLRP3 inflammasomes in microglia via CD36 receptors, which triggers the secretion of IL-1β and fosters a pro-inflammatory environment. α-synuclein also interacts with the lymphocyte-activation gene 3 receptor on astrocytes, inducing CXCL10 release and recruiting peripheral Th17 cells into the CNS, further exacerbating immune-mediated neuronal damage [73]. Autophagy and ubiquitin-proteasome system dysfunction create a vicious cycle: reduced clearance of α-synuclein aggregates leads to sustained microglial activation, preserving the release of pro-inflammatory cytokines (e.g., TNF-α, IL-6) and oxidative stress [74]. These inflammatory cytokines, in turn, inhibit autophagic flux, worsening α-synuclein accumulation. Furthermore, peripheral immune cells, such as infiltrating macrophages, contribute to proteostasis failure by releasing reactive oxygen species (ROS) and proteasome inhibitors (e.g., NO), further linking immune activation to neuronal proteinopathy. Cytokines like TNF-α, IL-1β, and IL-6, as well as the chemokine CCL2/MCP-1, are involved in this process, either directly damaging dopaminergic neurons or exacerbating inflammation [75].

In ALS, genetic mutations and protein abnormalities in neuronal cells, along with the intracellular stress responses they trigger, lead to the activation of microglia, which release inflammatory mediators that disrupt the BBB and permit CNS, triggering an autoimmune response [76,77]. Abnormalities in neuro-immune signaling pathways, including cytokines and chemokines, further exacerbate neuronal damage and death [78,79]. Common pathological phenomena in ALS include oxidative stress and mitochondrial dysfunction, particularly in motor neurons, leading to excessive production of ROS and consequent cellular damage by activation of microglia and astrocytes, exacerbating the inflammatory response [80]. Autophagy and UPS dysfunction contribute to the accumulation of abnormal proteins such as SOD1 and TDP-43, which can directly damage motor neurons or activate glial cells, intensifying inflammation [81,82].

## 6. Brain–Adipose Tissue Axis

While numerous axes linking the brain to abdominal organs have been extensively documented, the precise nature of the interaction between the brain and visceral white adipose tissue (vWAT) has only recently garnered significant scientific attention.

Adipose tissue, primarily white adipose tissue, serves as the central repository for energy in the form of triacylglycerols and plays a crucial endocrine role through hormone secretion. Its metabolic activity is highly sensitive to insulin, promoting lipogenesis; energy deprivation triggers lipolysis to supply free fatty acids and glycerol for energy production. The factors involved both in lipogenesis and in lipolysis can exert profound systemic effects, directly influencing distant organs, including the brain [83,84]. Adipose tissue secretes a range of signaling molecules known as adipocytokines, including leptin, ghrelin, adiponectin, resistin, and visfatin, which are also secreted by peripheral immune cells. In obesity, macrophages infiltrate adipose tissue and, together with adipocytes, create an inflammatory environment that serves as a rich source of adipocytokines. The subsequent activation of (adipo-)cytokine receptors on peripheral immune cells may trigger complications of metabolic syndrome through various immunological mechanisms [85]. The link between excessive adiposity and cognitive decline is attributed to systemic metabolic rewiring. A key molecular mechanism involves the NLRP3 inflammasome in visceral adipose tissue, which impairs cognitive function through the IL-1β-mediated activation of brain-resident microglia. This process is initiated by the secretion of IL-1β from local macrophages able to trigger a central inflammatory response [86].

**Figure 5:**
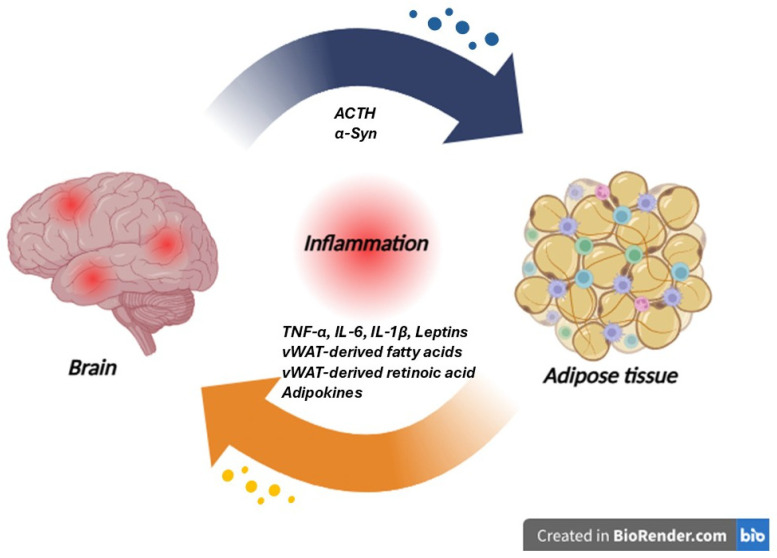
Schematic representation of the brain–visceral white adipose tissue (vWAT) axis in neurodegenerative diseases. The vWAT acts as an active endocrine and immunometabolic organ, releasing pro-inflammatory mediators such as TNF-α, IL-6, IL-1β, leptin, vWAT-derived fatty acids, vWAT-derived retinoic acid, and other adipokines. These circulating factors can exacerbate systemic inflammation, cross the blood–brain barrier, and promote glial activation, neuroinflammation, and neuronal dysfunction in the central nervous system. Conversely, brain-derived signals, including ACTH and α-synuclein (α-Syn), can act on adipose tissue, further modulating its inflammatory and metabolic activity. This reciprocal crosstalk establishes a vicious cycle in which peripheral metabolic dysfunction amplifies neuroinflammatory processes, contributing to the pathophysiology of neurodegenerative diseases (the image was created with BioRender.com online software: https://www.biorender.com).

In Figure 5, a schematic representation of the brain–visceral white adipose tissue (vWAT) axis is reported.

The brain–visceral white adipose tissue axis is increasingly recognised for its potential involvement in the pathogenesis of AD. The vWAT, as an active endocrine organ, releases various factors that can directly impact brain health and contribute to AD-related pathology [86]. Key mechanisms include the release of vWAT-derived fatty acids, which can cross the BBB and induce neuroinflammation and neuronal dysfunction. Furthermore, the immunological properties of vWAT, characterized by the secretion of pro-inflammatory cytokines and adipokines, contribute to systemic inflammation that can exacerbate neuroinflammation and amyloid-beta deposition in the brain [87,88]. Dysregulation of vWAT-derived retinoic acid, which plays a role in neuronal development and plasticity, may also contribute to cognitive decline. Finally, vWAT-regulated insulin resistance is a critical condition, as peripheral insulin resistance is a well-established risk factor for AD, leading to impaired brain glucose metabolism and insulin signaling, which are hallmarks of the disease [89,90]. Adipose tissue-derived mesenchymal stem cells (ADSCs) have been shown to improve cognitive function in ageing, although in the context of obesity, they can be either pro-inflammatory, by inducing T cell proliferation, or anti-inflammatory. Transplanting ADSCs has been observed to ameliorate cognitive impairments, enhance Aβ clearance, and suppress neuroinflammation by modulating systemic and central SIRT1 levels. Furthermore, intraventricular administration of ADSCs can improve AD pathogenesis by influencing central cholesterol homeostasis. Collectively, these findings highlight the role of the brain–adipose axis in cognitive regulation, with changes in the adipose tissue milieu contributing to cognitive decline and bridging the gap between metabolic and neurological disorders [91,92]. Understanding these intricate interactions provides novel insights into the systemic contributions to AD and opens avenues for potential therapeutic interventions targeting metabolic and inflammatory pathways.

The intricate relationship between PD and adipose tissue metabolism is an area of growing research, highlighting how peripheral metabolic states can influence neurodegeneration. Epidemiological studies suggest a complex link, with some indicating that obesity may increase PD risk, while paradoxically, most PD patients exhibit lower body weight than healthy individuals, which can partially recover with effective treatments [93].Adipose tissue, particularly brown adipose tissue (BAT), has been implicated in PD pathology. In 6-hydroxydopamine (6-OHDA)-induced PD models, thermogenic interscapular BAT (IBAT) is activated, leading to increased triglyceride decomposition and heat production [94]. Furthermore, in transgenic PD models, A53T mice overexpressing human αSyn, early αSyn accumulation in hypothalamic orexin neurons is associated with a later reduction in overall fat mass and an increase in energy expenditure [95]. Similarly, Thyl-αSYN transgenic mice also display adiposity loss, altered feeding behaviour, and reduced energy expenditure [96].

Adipose tissue also plays a crucial role through the secretion of adipokines, such as leptin. Leptin, a hormone produced by adipose tissue, signals the brain about body fat levels to regulate appetite and metabolic rate. Studies have shown that PD patients experiencing body weight loss often exhibit lower plasma leptin levels and reduced adipose tissue content [97]. Conversely, some patients undergoing deep-brain stimulation for PD demonstrate weight gain, potentially linked to increased leptin levels and leptin resistance. The impact of adipose tissue extends to inflammatory processes, where free fatty acids in obese individuals can activate astrocytes, promoting the release of pro-inflammatory factors that contribute to neuronal damage.

Dysregulation of lipid metabolism and adipose tissue dynamics pathways has recently been recognized also in ALS [98]. While a reduction in total fat mass has been consistently observed in ALS patients and animal models, the distribution and specific characteristics of adipose tissue appear critical [99,100]. Investigations into body fat distribution using MRI have revealed an increase in visceral fat alongside a non-significant trend of decreased subcutaneous fat in ALS patients. This distinction is clinically relevant, as subcutaneous fat, indicative of energy storage, has been correlated with prolonged survival in male ALS patients, suggesting a protective role. Conversely, elevated visceral fat, often associated with insulin resistance and systemic inflammation, may contribute to disease progression [100]. Furthermore, a shift towards a hypermetabolic state in ALS is supported by observations of elevated lipolysis and the “browning” of WAT (formation of beige adipocytes with thermogenic properties) in SOD1^G93A^ mouse models [101].

## 7. Muscle–Brain Axis

The muscle–brain axis has emerged as a critical area of investigation in the field of neurodegeneration, highlighting the profound impact of skeletal muscle on central nervous system function and vice versa. This axis comprises a complex interplay of anatomical structures, physiological processes, and molecular signaling that involves the muscle as a motor effector and the brain as the central control.

**Figure 6:**
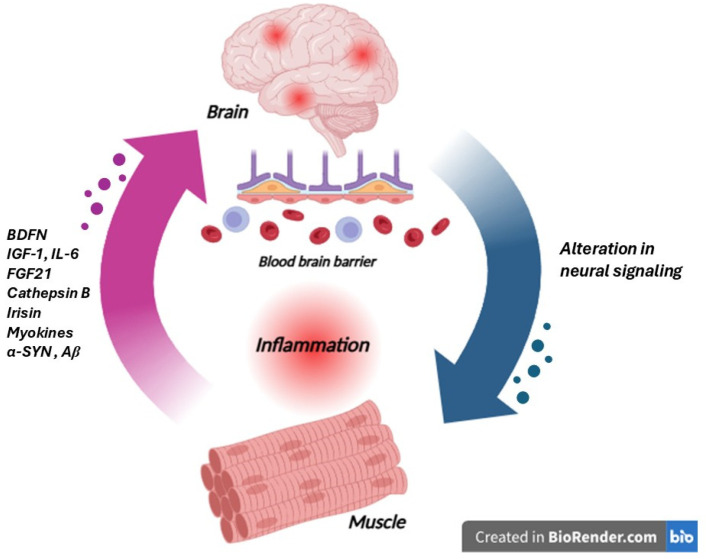
Schematic representation of the muscle–brain axis in neurodegenerative diseases. Skeletal muscle functions as an active endocrine organ, releasing myokines and exercise-induced factors such as BDNF, IGF-1, IL-6, FGF21, cathepsin B, and irisin, which modulate brain function, neuroinflammation, and synaptic plasticity. BDNF enhances neurogenesis and cognitive resilience, while IGF-1 promotes neuronal survival and reduces inflammatory signaling. IL-6, although classically pro-inflammatory, exerts anti-inflammatory and neuroprotective effects when released in response to muscle activity. FGF21 contributes to metabolic regulation and exerts neuroprotective actions by reducing amyloid-β accumulation. Cathepsin B and irisin have been implicated in memory improvement and synaptic regulation. However, under pathological conditions, muscle can also release misfolded proteins such as α-synuclein (α-Syn) and Aβ, which may cross the blood–brain barrier and exacerbate neurodegeneration. This bidirectional interaction is further amplified by inflammation and alterations in neural signaling, highlighting the role of the muscle–brain axis in neurodegenerative disorders (the image was created with BioRender.com online software: https://www.biorender.com).

The peripheral component of the muscle–brain axis is principally constituted by skeletal muscle tissue, histologically characterized by multinucleated muscle fibers, organized into fascicles, vascularized, and innervated [102]. The principal function is to generate movement, but it is also able to release molecules termed myokines, able to act locally, with paracrine activity, and distally, with endocrine activity, and to exert a wide range of effects on various tissues and organs, including the brain. The central component of this axis encompasses various brain regions, including the hippocampus, prefrontal cortex, cerebellum, and basal ganglia, areas crucial for cognitive function, motor control, and neuroplasticity. These brain regions are equipped with receptors capable of binding circulating myokines, enabling direct molecular communication from muscle to the central nervous system. Furthermore, the brain exerts control over muscle function through the somatic motor nervous system, promoting and modulating muscle contraction. The common myokines are brain-derived neurotrophic factor (BDNF), insulin-like growth factor-1 (IGF-1), fibroblast growth factor 21 (FGF21), interleukin-6 (IL-6), irisin, and cathepsin B, which have been shown to cross the blood–brain barrier or act on circumventricular organs to influence brain function [103] as reported in Figure 6. For example, BDNF plays a critical role in neurogenesis, synaptic plasticity, and cognitive function, and its circulating levels are positively correlated with physical activity and muscle mass. IGF-1, another key myokine, promotes neuronal survival and growth. While IL-6 is traditionally known as a pro-inflammatory cytokine, its release during exercise exhibits anti-inflammatory effects, promoting neuroprotection. Irisin, a cleaved fragment of fibronectin type III domain-containing protein 5 (FNDC5), has garnered significant attention for its ability to induce the expression of BDNF in the hippocampus, potentially contributing to the cognitive benefits of exercise. Cathepsin B, a lysosomal cysteine protease released by muscle, has also been implicated in enhancing neurogenesis and memory function [104].

Furthermore, systemic inflammation, often influenced by muscle health and physical activity levels, can indirectly impact the brain by modulating neuroinflammation and glial cell activation. Conversely, the brain influences muscle through neural signals, regulating muscle protein synthesis, energy metabolism, and fiber type adaptation in response to activity demands [105]. Aberrant neural signaling, as seen in neurological disorders, can lead to muscle atrophy and weakness, further highlighting the bidirectional nature of this axis [106]. Understanding the intricate molecular mechanisms underlying the muscle–brain axis holds significant promise for developing novel therapeutic strategies targeting both muscle and brain health in various physiological and pathological conditions, including neurodegenerative diseases. The mechanisms by which myokines exert their neuroprotective effects are multifaceted: they can directly cross the BBB and interact with neuronal and glial cells, modulating synaptic plasticity, promoting neurotrophic factor signaling, and enhancing neuronal survival, and can indirectly influence the CNS by modulating peripheral inflammation, improving metabolic health, and altering the gut microbiome [107].

In the context of AD, myokines have shown promising neuroprotective effects. Exercise, a potent stimulus for myokine release, has been consistently associated with a reduced risk of cognitive decline and AD [107]. Increased circulating levels of BDNF following exercise have been linked to improved cognitive performance and reduced Aβ pathology in preclinical models [108]. Irisin, another exercise-induced myokine, has also demonstrated neuroprotective properties by promoting neurotrophic factor expression and reducing neuroinflammation in AD models [109]. Furthermore, FGF21, while primarily known for its metabolic effects, has shown potential in mitigating Aβ burden and improving cognitive function in AD transgenic mice [110].

Myokines have been demonstrated to exert a beneficial effect also in PD [111]. Exercise is a well-established non-pharmacological intervention for PD, improving motor and non-motor symptoms, as myokines released during exercise may contribute to these benefits by modulating neuroinflammation and oxidative stress, key pathological processes in PD [107]. For instance, IL-6, while traditionally considered a pro-inflammatory cytokine, exhibits anti-inflammatory effects in the CNS at certain concentrations and can be induced by exercise [104]. In preclinical studies, cathepsin B, another myokine, has been shown to promote the clearance of α-synuclein, the main protein aggregate in PD [112].

In ALS, the role of the muscle–brain axis is not yet well specified, and it can be inferred from the general mechanisms described for other neurodegenerative diseases [103]. ALS, characterized by the progressive degeneration of motor neurons, leading to muscle atrophy and paralysis, inherently involves a compromised muscle–brain connection [113]. The general principles of myokine action, such as modulation of neuroinflammation, oxidative stress, and neurotrophic support, are highly relevant to ALS pathology. Given that myokines influence neuronal survival, synaptic plasticity, and metabolic health, it is hypothesized that a healthy and active muscle–brain axis could exert protective effects [114]. The observed benefits of exercise in mitigating neurodegenerative processes in AD and PD, mediated by myokines, suggest a similar potential in ALS [104,105]. Further research is needed to specifically elucidate the roles of individual myokines and the impact of the muscle–brain axis dynamics in the context of ALS pathogenesis and progression.

## 8. Conclusions

In the development and advancement of neurodegenerative disease, the pathological processes are not limited to a single organ, but an intricate and closely connected interaction between multiple organ systems collectively shapes the progression of illness. Several “axes” are involved, including the gut–brain, liver–brain, adipose-brain, muscle–brain, bone-brain, and gut-microbiota-brain axes, all of which communicate with the brain through specific pathways and influence and regulate one another. The emerging concept of inter-organ communication highlights the critical role of peripheral organs in the pathogenesis and progression of neurodegenerative diseases. The intricate bidirectional signaling between the brain and peripheral organs, such as the gut, liver, adipose tissue, muscle, and immune system, contributes significantly to neuroinflammation, protein misfolding, and neuronal dysfunction.

Disruption of these inter-organ crosstalk pathways can exacerbate disease pathology by amplifying systemic inflammation and affecting the homeostasis of key metabolic and immune processes. For example, an imbalance in the gut microbiota, such as an overgrowth of harmful bacteria, can compromise the intestinal barrier. This allows inflammatory agents such as TNF-α and IL-6 to enter the bloodstream, triggering a widespread inflammatory response. These cytokines then activate the JAK/STAT signaling pathway in the liver, which inhibits detoxifying enzymes like cytochrome P450, with a consequent release of neurotoxins, and alteration of lipid metabolism, ultimately damaging the membranes of dopamine-producing neurons in the brain. Furthermore, TNF-α can harm blood vessel linings, reducing the production of nitric oxide, which decreases vascular elasticity and can lead to autonomic nervous system dysfunction and orthostatic hypotension. These pathological processes are interconnected by common mechanisms like neuroinflammation (via the NF-κB pathway), oxidative stress (due to ROS accumulation), and mitochondrial dysfunction. For instance, ROS produced by poorly functioning mitochondria in skeletal muscles can circulate, intensifying oxidative damage in the lungs and further compromising the intestinal barrier, creating a detrimental loop among the muscle, lung, and gut axes. Moreover, excess iron resulting from liver metabolic issues can activate osteoclasts in bone tissue via the RANKL pathway, while also stimulating microglial cells in the brain, thereby worsening neuroinflammation. Myokines released by muscles during physical activity provide neuroprotective effects, indicating the potential of exercise as a therapeutic intervention. Moreover, the immune system acts as a central mediator across these axes, where peripheral immune cell infiltration into the central nervous system accelerates neurodegeneration.

These inter-organ interactions present potential application in the biomarkers field for early diagnosis and therapeutic targets, emphasizing the importance of peripheral inflammation and metabolic regulation in neurodegenerative disease personalized treatment.

**Figure 7:**
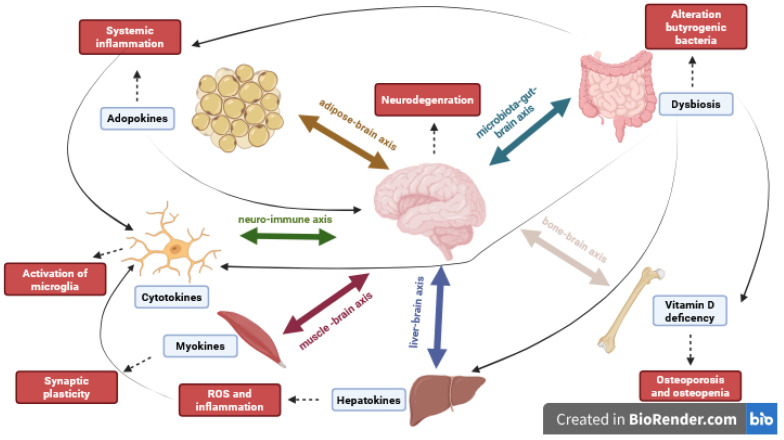
Crosstalk between axes and their synergistic effects on neurodegenerative disease. The different axes have bidirectional communication with the brain, and the molecules released by the organs have effects not only on the brain but also interfere with other axes, confirming the multisystemic nature of neurodegenerative diseases, which are mainly driven by inflammatory molecules.

In Table 1 and Figure 7 a summary of interplay of different pathways in neurodegenerative diseases is reported.

Further research is essential to fully elucidate the precise mechanisms underlying these communication pathways and to explore therapeutic strategies targeting peripheral organs to mitigate the progression of neurodegenerative diseases, also in consideration of the importance of a personalized medical approach.

Considering these findings, a holistic approach that integrates both central and peripheral systems is necessary for the prevention, diagnosis, and management of NDs, offering novel avenues for therapeutic intervention.

## Figures and Tables

**Table 1:** The Interplay of different brain axes in neurodegenerative disease.

	*Microbiota–Gut–Brain Axis*	*Brain–Gut–Bone Axis*	*Liver Brain Axis*	*Neuro-Immune Axis*	*Brain–Adipose Tissue Axis*	*Muscle–Brain Axis*
***Gut–brain axis***	LPS, amyloid, and other toxins mediate systemic inflammation [21,23,31,37]			LPS, amyloid, and other toxins mediate systemic inflammation [21,23,31,37]		
***Microbiota–gut–brain axis***	Dysbiosis causes misfolding or aggregation of protein [29,30,31]	Osteoporosis and osteopenia [38,39,40]	SCFAs, bile acids, and lipopolysaccharides impact neuroinflammation [48,49]	Dysbiosis activates microglia, promoting an inflammatory [29,30,31,32,33,34]	Dysbiosis reduces butyrate production and other adipokine secretion [88,89,90]	
***Brain–gut–bone axis***					Vitamin D deficiency reduces butyrate production and other adipokine secretion [38,39,40]	
***Liver brain axis***	SCFAs, bile acids, and lipopolysaccharides impact neuroinflammation [48,49]			Hepatic fatty acid metabolism and detoxification capacity may intensify systemic inflammation [50,51,52,53,54,55]		
***Neuro-immune axis***			Hepatokines promote oxidative stress and inflammation [48,49]		TNF-α, IL-1β, and IL-6, and chemokines like CCL2/MCP-1 and CXCL10, promoting neuroinflammation and recruiting peripheral immune cells [59,60,61,62,63]	Myokines modulate synaptic plasticity and cognitive function [103,104]

## Data Availability

No new data were created or analyzed in this study.

## Abbreviations

The following abbreviations are used in this manuscript:

ND	Neurodegenerative diseases
AD	Alzheimer’s disease
PD	Parkinson’s disease
ALS	Amyotrophic Lateral Sclerosis
TNF-α	Tumor Necrosis Factor-alpha
IL-6	Interleukin-6
IL-1β	Interleukin-1 beta
FGF21	Fibroblast growth factor 21
BDNF	Brain-derived neurotrophic factor
HSPs	Heat shock proteins
ACTH	Adrenocorticotropic Hormone
CNS	Central nervous system
ENS	Enteric nervous system
ANS	Autonomic nervous system
NTS	Nucleus of the solitary tract
GM	Gut microbiota
LPS	Lipopolysaccharide
TLRs	Toll-like receptors
SOD1	Superoxide dismutase 1
BMD	Bone mineral density
BBB	Blood–brain barrier
UPS	Ubiquitin-proteasome system
ROS	Reactive oxygen species
vWAT	Visceral white adipose tissue
BAT	Brown adipose tissue
